# The yin and yang of 15-lipoxygenase-1 and delta-desaturases: Dietary omega-6 linoleic acid metabolic pathway in prostate

**DOI:** 10.1186/1477-3163-5-9

**Published:** 2006-03-27

**Authors:** Uddhav Kelavkar, Yan Lin, Doug Landsittel, Uma Chandran, Rajiv Dhir

**Affiliations:** 1 Department of Urology and University of Pittsburgh Cancer Institute, 5200 Center Ave., SHMC-Suite G-37, Pittsburgh, PA, 15232, USA; 2 Department of Biostatistics, Graduate School of Public Health, University of Pittsburgh, 130 DeSoto Street, Pittsburgh, PA, 15261, USA; 3 Department of Mathematics and Computer Science, Duquesne University, Pittsburgh, PA, 15282, USA; 4 Department of Pathology and University of Pittsburgh Cancer Institute, 5200 Center Ave., Pittsburgh, PA, 15232, USA

## Abstract

One of the major components in high-fat diets (Western diet) is the omega (ω, n)-6 polyunsaturated fatty acid (PUFA) called linoleic acid (LA). Linoleic acid is the precursor for arachidonic acid (AA). These fatty acids are metabolized to an array of eicosanoids and prostaglandins depending upon the enzymes in the pathway. Aberrant expression of the catabolic enzymes such as cyclooxygenases (COX-1 and/or -2) or lipoxygenases (5-LO, 12-LO, 15-LO-1, and 15-LO-2) that convert PUFA either AA and/or LA to bioactive lipid metabolites appear to significantly contribute to the development of PCa. However, PUFA and its cellular interactions in PCa are poorly understood. We therefore examined the mRNA levels of key enzymes involved in the LA and AA pathways in 18 human donor (normal) prostates compared to 60 prostate tumors using the Affymetrix U95Av2 chips. This comparative (normal donor versus prostate cancer) study showed that: 1) the level of 15-LO-1 expression (the key enzyme in the LA pathway) is low (*P *< 0.001), whereas the levels of delta-5 desaturase (*P *< 0.001, the key enzyme in the AA pathway), delta-6 desaturase (*P *= 0.001), elongase (*P *= 0.16) and 15-lipoxygenase-2 (15-LO-2, *P *= 0.74) are higher in donor (normal) prostates, and 2) Contrary to the observation in the normal tissues, significantly high levels of only 15-LO-1; whereas low levels of delta-6 desaturase, elongase, delta-5 desaturase and 15-LO-2 respectively, were observed in PCa tissues. Although the cyclooxygenase (COX)-1 and COX-2 mRNA levels were high in PCa, no significant differences were observed when compared in donor tissues. Our study underscores the importance of promising dietary intervention agents such as the omega-3 fatty acids as substrate competitors of LA/AA, aimed primarily at high 15-LO-1 and COX-2 as the molecular targets in PCa initiation and/or progression.

## Findings

**Figure 1:**
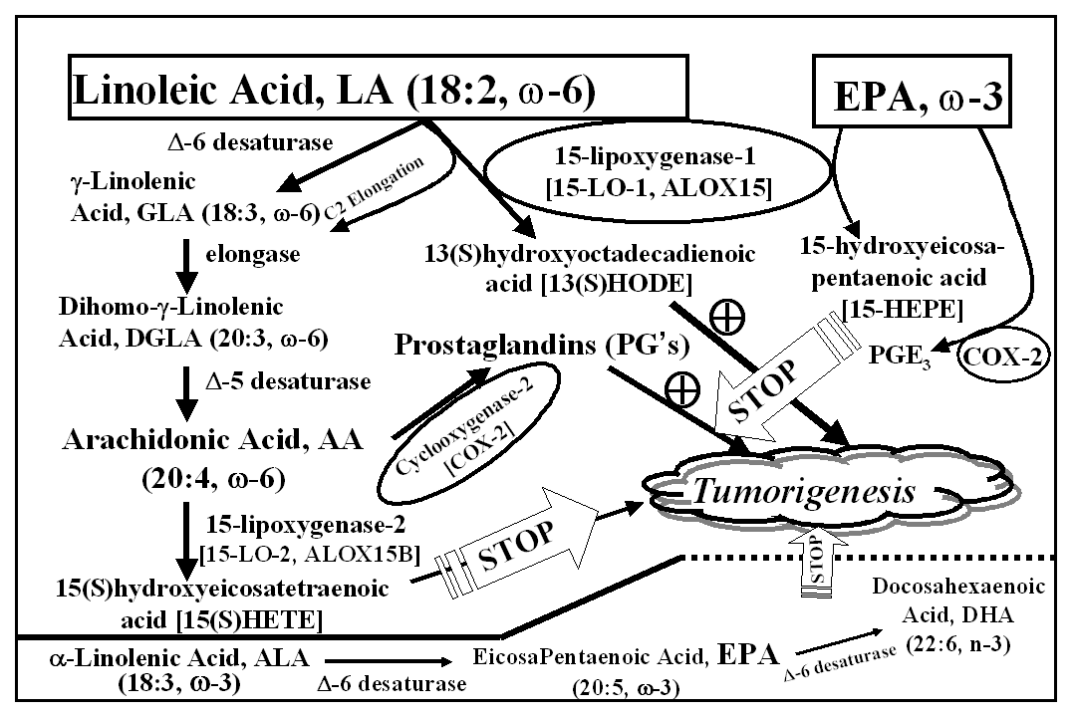
Omega-6 and -3 fatty acids & Prostate Cancer.

**Figure 2:**
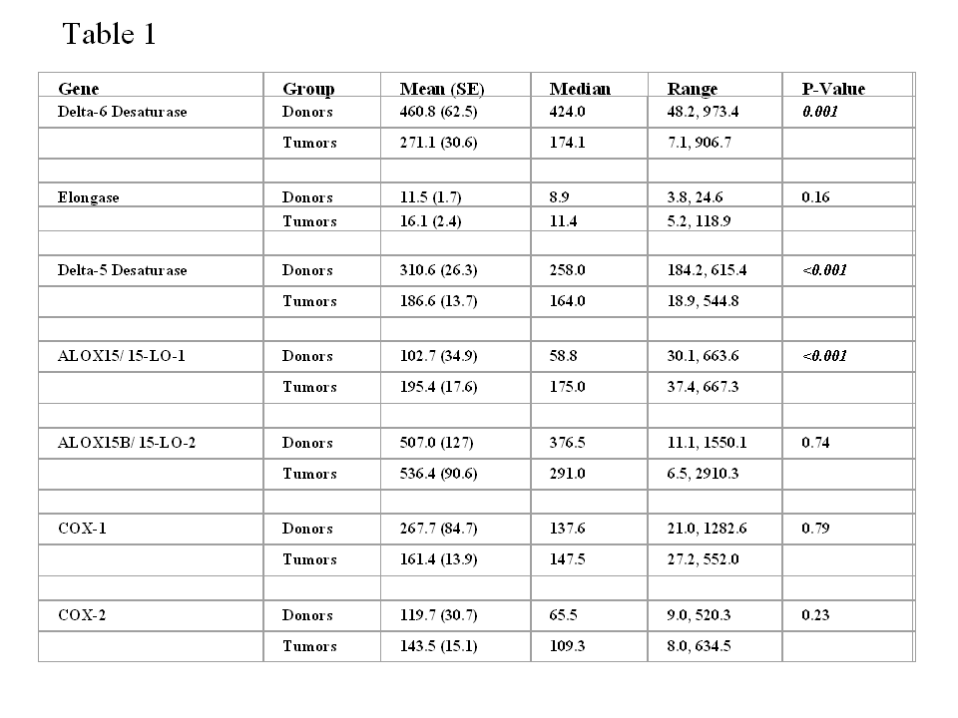
r

Prostate cancer (PCa) remains the second most commonly diagnosed cancer in American men, with over 230,000 new cases predicted for 2005 alone [1]. Projections estimate that the number of new cases of PCa during the next 20 years will more than double and the number of men dying of the disease could double or triple during this same interval [2]. The incidence and mortality of PCa vary greatly in different geographic regions of the world [3,4]. Lifestyle factors, such as diet, likely contribute to these differences since migrants from low- to high-risk regions typically develop a higher PCa risk, often within one generation. The typical U.S. diet-rich in vegetable oils, animal fats and meats that contain heterocyclic aromatic amine carcinogens [5-8] and poor in fruits and vegetables that contain carcinogen-detoxification enzyme inducers and antioxidants such as lycopene [6,9,10]-almost certainly plays a role in the high incidence of PCa found in America. Examples of dietary fat that impact PCa include n-3 and n-6 polyunsaturated fatty acids (PUFAs), both of which play important roles in many normal human biological processes. As humans cannot synthesize sufficient quantities of n-3 and n-6 PUFAs, they are considered essential fatty acids. While all mammalian cells can interconvert the PUFAs within each series by elongation, desaturation and retro conversion, the two series are not interchangeable due to the lack of the Fat-1 gene [11], which encodes the n-3 desaturase enzyme [12,13]. Linoleic acid (LA; 18:2, n-6) represents an n-6 PUFA commonly found in high-fat Western diets [14]. Terrestrial plants synthesize LA, and once ingested by mammals, LA is either metabolized to 13-(S)-hydroxyoctadecadienoic acid [13-(S)-HODE] or converted further to arachidonic acid (AA; 20:4, n-6). Three fatty acids, found primarily in fish oils, comprise the n-3 family: Alpha linolenic acid (ALA or α-LNA; 18:3, n-3), eicosapentaenoic acid (EPA; 20:5, n-3), and docosahexaenoic acid (DHA; 22:6, n-3). ALA, synthesized by cold-water vegetation, is converted to EPA and DHA by fish. Of note, through the same series of enzymes used to convert LA to AA, humans can synthesize EPA from ingested ALA. EPA can then be converted to DHA. We are left with tantalizing suggestions from epidemiological investigations that consuming n-3 fatty acids have many health benefits [15-20]. Complimentary Alternative Medicine (CAM) involving the use of natural and dietary agents for cancer prevention may prove a valuable strategy in the fight against PCa [21-33]. Due to the probable impact of n-3 and n-6 PUFAs on PCa development, delaying the PCa progression by n-3 PUFAs will provide for tangible benefits in alleviating the adverse consequences of PCa, which is approaching epidemic proportions in the United States. Therefore it is imperative to understand how the n-6 and n-3 pathways operate in human prostate especially during prostate carcinogenesis. We analyzed the mRNA expression levels of these enzymes in normal and cancer prostate tissues from humans to study the LA and AA pathways. The tumor and normal donor tissue samples were acquired from the University of Pittsburgh Medical Center under stringent Institutional Review Board guidelines with appropriate informed consent. Specimens were received directly from the operating room. Samples (>500 mg) were excised and snap frozen in liquid nitrogen within 30 min of excision and stored at -80°C in the University of Pittsburgh Pathology Tissue Bank until extraction of RNA. All samples were submitted for pathology evaluation. In every case, the tissue was excised from the junction between the ejaculatory duct and the prostatic urethra in the peripheral zone of the prostate and their histological diagnosis was confirmed microscopically. Donor tissue specimens were received through a collaborative arrangement with the Center for Organ Recovery and Education (CORE), the local organ procurement agency. The arrangement allows the University of Pittsburgh Pathology Tissue Bank to acquire normal prostates and associated serum/plasma specimens from healthy individuals who have donated their organs for transplant. There is extensive collaborative support from CORE. The donor prostatectomies harvested from brain dead, perfused donors and are bathed in Ringer's Lactate solution and transported on wet ice. These donor prostates are transported and handled with the harvested "transplant" organs. This significantly reduces transit time and minimizes the degradation of RNA. The processing methodologies used consist of snap freezing tissues in bulk, freezing in OCT and processing the tissues for routine histology (paraffin embedded tissues). For microarray analysis, the donor samples were excised from the same zone as the tumor samples. The 60 tumor samples used in this study consisted of 2, 13, 27, 6 and 12 cases of primary prostatic adenocarcinoma of Gleason grade 5, 6, 7, 8 and 9 respectively. There were 4, 20, 23 and 13 cases spanning the age groups 40–49, 50–59, 60–69 and 70–79 respectively. Of the cases, 36 were stage T3 or higher with 2, 22, 23, 11 and 2 cases of stage T2a, T2b, T3a, T3b and 4 respectively. Of the donors, 11 are under and 7 are over the age of 40. Complementary RNA (cRNA) were prepared and hybridized to Affymetrix oligonucleotide arrays as previously described [34]. Log transformed prostate tissue expression levels of a panel of seven key enzymes involved in the LA and AA pathways were compared between the donors and prostate cancer patients using two sample t-test. Seven individual probe sets were available for the Δ-5 Desaturase. The test was done on the mean log expression levels of these 6 probe sets. Descriptive summary statistics were presented for each of the 7 genes within both, the donor and tumor groups. Differences in gene expressions were evaluated using both t-tests of log-transformed data and the Wilcoxon rank-sum test. Log-transformations were utilized since data were relatively skewed. Rank-sum tests, which are the non-parametric analog to t-tests and based on the ranks of the data were utilized to assure that any violations of the assumptions for the t-test did not critically affect results particularly by multiple comparisons, as they would remain unchanged even after using the conservative Bonferroni adjustment (which is comparing the P-value to 0.05 divided by the number of comparisons). We examined the mRNA levels of seven key enzymes involved in the LA and AA pathways in microdissected, 18 human donor (normal) prostates compared to 60 prostate tumors (Gleason grades 8–10) by Affymetrix oligonucleotide array (Table 1 - see Figure 2). Comparison of the gene expressions using t-test after log transformation as well as by rank-sum test suggests that the differences, wherever observed, are statistically highly significant. Only the log-transformed t-test results are reported in this study since the rank-sum test gave the same results. The results were not affected by multiple comparisons, as they remain unchanged even after using the conservative Bonferroni adjustment (which is comparing the *P*-value to 0.05 divided by the number of comparisons). However, the analyses conducted in the present study do not evaluate the prognostic ability of these genes, they merely evaluate whether they are differentially expressed. Our data on donors spanned from ages 5 to 60 and all tumor patients are older than 40. Therefore, in order to examine whether or not the differential gene expression between donors and patients is affected by age specific differences in their prostates, we segmented the donors into different age groups and compared only the 40 to 60 year old donors with tumors of the same age group. Although the number of cases in the study were small, the expression pattern observed in this age matched analysis is identical (data not shown) to that when all donors are included suggesting that potential age related differences in donor prostates do not contribute to the results of the donor versus tumor analysis. This comparative (normal donor versus prostate cancer) study showed that: 1) the level of 15-LO-1 expression (the key enzyme in the LA pathway) is low (P < 0.001), whereas the levels of delta-5 desaturase (P < 0.001, the key enzyme in the AA pathway), delta-6 desaturase (P = 0.001), elongase (P = 0.16) and 15-lipoxygenase-2 (15-LO-2, P = 0.74) are higher in donor (normal) prostates, and 2) However in PCa tissues, significantly high levels of only 15-LO-1; whereas low levels of delta-6 desaturase, elongase, delta-5 desaturase and 15-LO-2 respectively, were observed. Although the cyclooxygenase (COX)-1 and COX-2 mRNA levels were high in PCa, no significant differences were observed when compared in donor tissues. This study suggests that an increase in the n-6 to n-3 PUFA ratio in diet when the n-6 enzymes such as 15-LO-1 and COX-2 are aberrantly expressed can promote PCa, and the need for designing promising early dietary intervention agents such as the omega-3 fatty acids as substrate competitors of LA/AA, aimed primarily at high 15-LO-1 and COX-2 as the molecular targets (Figure 1). Early introduction of "good" metabolites by dietary n-3 PUFA thereby reducing the "bad" metabolites of dietary n-6 can slow down or even inhibit PCa development in individuals with aberrant 15-LO-1 and COX-2 expressions. Thus, by combining a reduction of dietary n-6 fat with an intake of n-3 fatty acids would be more attainable and particularly beneficial to the approximately 80 million baby boomers that will begin turning 60 years of age in 2 years [2], and those who follow.

## Abbreviations

13-S-HODE, 13-S-hydroxyoctadecadienoic acid; 15-LO, 15-lipoxygenase; COX, cyclooxygenase; LA, linoleic acid; AA, arachidonic acid; PG, prostaglandin; PCa, prostatic carcinoma; AA, arachidonic acid; COX, cyclooxygenase; LO, lipoxygenase; HETE, hydroxyeicosatetraenoic acid.

## Competing interests

The author(s) declare that they have no competing interests.

## Authors' contributions

UK conceived the study, carried out the data query analysis and drafted the manuscript. YL and DL performed the statistical analysis. UC carried out the data retrieval and query analysis. RD participated in its design. All authors read and approved the final manuscript.
